# Use of Carbon Nanotubes (CNTs) with Polymers in Solar Cells

**DOI:** 10.3390/molecules191117329

**Published:** 2014-10-28

**Authors:** Huda A. Alturaif, Zeid A. ALOthman, Joseph G. Shapter, Saikh M. Wabaidur

**Affiliations:** 1 Centre for NanoScale Science & Technology (CNST), Flinders University of South Australia, Bedford Park, Adelaide, SA 5042, Australia; E-Mails: altu0027@flinders.edu.au (H.A.A.); joe.shapter@flinders.edu.au (J.G.S.); 2 Advanced Material Research Chair, Chemistry Department, College of Science, King Saud University, Riyadh 11451, Saudi Arabia; E-Mail: swabaidur@ksu.edu.sa

**Keywords:** solar cells, carbon nanotubes, CNTs, organophotovoltaics, OPVs, polymers

## Abstract

There is a clear need to make energy cheap, readily accessible and green, while ensuring its production does not contribute to further climate change. Of all the options available, photovoltaics offer the highest probability of delivering a meaningful and sustainable change in the way society produces its energy. One approach to the development of such photovoltaics involves the use of polymers. These systems offer the advantages of cheap production, flexibility (and hence a range of deployment opportunities) and tunability of light absorption. However, there are issues with polymer-based photovoltaic systems and one significant effort to improve these systems has involved the use of carbon nanotubes (CNTs). This review will focus on those efforts. CNTs have been used in virtually every component of the devices to help charge conduction, improve electrode flexibility and in some cases as active light absorbing materials.

## 1. Introduction

Due to the need to produce green sources of energy, there has been a keen interest in finding solutions for improving efficiencies in solar cells. The most common cells in use commercially today are silicon-based solar cells. The high conversion efficiency (up to 25% in the lab), stability of high purity silicon, excellent charge transport properties and the mature processing technologies have led to silicon dominating the photovoltaic (PV) market [1,2]. However, the manufacturing process of high efficiency silicon solar cells suffers from low throughput and thus these solar cells are costly, preventing silicon PV from contributing significantly as a source in the world’s energy. In order to solve some of these issues, thin film solar cells, such as CdTe, CuIn_x_Ga_1–x_Se_2_ (CIGS), Cu_2_ZnSnS_4_ (CZTS) and thin film silicon have become the subject of intense research [2,3]. The heavy metal Cd is toxic and the metal is expensive, thus limiting the development of CdTe and CIGS-based solar cells, respectively [4,5]. The low cost material CZTS was recently introduced and it is still underdeveloped [6]. For thin film silicon solar cell technology, the low efficiency and instability from the Staebler-Wronski effect restrict its usage as a solar cell [2]. The tradeoff between the cost and the performance of these solar cells is still a great barrier to wide scale commercial application. Therefore, it becomes essential to search for alternative materials. The possible candidates are organic material-based solar cells and organic-inorganic hybrid solar cells.

Organic materials have both conducting and semiconducting properties which are really promising for optoelectronic devices [7]. For solar cells, organic materials are attractive due to their potential low cost, simple manufacturing process and high throughput, suggesting that organic materials have the potential to make a major impact on the PV market. The absorption coefficient of organic semiconductors is very high which allows light to absorb within a very thin layer leading to low cost solar cells [8]. In addition, the efficiency of organic solar cells increases with temperature, while most conventional inorganic solar cells lose efficiency with increasing temperature [9]. Different organic materials including organic molecules, conjugated polymers and four typical carbon materials (e.g., amorphous carbon, fullerenes, CNTs and graphene) are used for both organic and organic-inorganic hybrid solar cells [7,10,11]. Among these, recent research on CNTs indicates it is a potential material for the organic and hybrid solar cells. Moreover, CNTs exhibit interesting optoelectronic, physical and chemical properties, required for many viable applications [12,13], although large scale commercial production will still need the development of robust CNT sorting and handling protocols. Investigation of single-wall carbon nanotube (SWCNT)–polymer solar cells has been conducted towards developing alternative, lightweight, flexible devices for space power applications.

Solar cells have undergone considerable development over the past two decades from first generation silicon (Si) solar cells [14] to second generation solar cells based on semiconductor thin films [15] to recent development of third generation solar cells represented by dye sensitized solar cells (DSSCs), in some cases referred to as hybrid cells, and organic semiconductor solar cells or organophotovoltaic (OPV) cells [16,17]. The efficiencies of OPVs have tripled in recent years [18]. One of the areas of development of these cells has been the addition of carbon nanotubes to the various components of the devices. This review will focus on the incorporation of CNTs, their role in the cells and their ultimate effectiveness.

## 2. Overview of Carbon Nanotubes

Carbon nanotubes (CNTs) are composed of hexagonally oriented carbon atoms with a cylindrical nanostructure. CNTs have been constructed with extremely high length-to-diameter ratio (up to 132,000,000:1) [19,20,21], which can be an important advantage for various applications including electronics, optics or sensing. CNTs are the strongest and stiffest materials yet discovered in terms of tensile strength and elastic modulus, respectively [22]. It has been shown that CNTs have a tensile strength 16 times higher than stainless steel. Composites of nanotubes and polymers have been shown to enhance the strength of the polymer considerably [23] which will be important in maintaining the flexibility of an operational OPV. The bonding structure is composed of *sp*^2^ bonds provides CNTs with this unique strength. The length of these tubular fibers varies from nanometers to thousands of micrometers providing an extremely high aspect ratio. Due to this high aspect ratio, CNTs have been proven to be excellent thermal and electrical conductors that can be used in various applications [24]. The thermal conductivity is five times higher than copper and will be important to enhance OPV cell lifetime by reducing degradation.

### 2.1. Structure and Classification of CNTs

CNTs can be classified as metallic or semiconducting depending on their diameter, arrangement of hexagon rings and tube length. The chirality of nanotubes, that is the way of wrapping the graphene layer to make the tube, determines the electrical characteristics of the nanotubes. The mechanical strength of CNTs can be strongly affected by the arrangement of the carbon atoms and the general defectiveness [20]. CNTs can be classified into two types depending on the how many graphene layers they have. Nanotubes consisting of round roll of a single layer are called single-walled CNTs (SWCNTs) and where there are a number of rolled layers, these nanotubes are referred to as multi-walled CNTs (MWCNTs).

### 2.2. Single-Walled Carbon Nanotubes (SWCNTs)

The structure of a SWCNT can be identified by wrapping a one-atom-thick layer of graphite called “graphene” into a cylinder. The way the graphene is wrapped is given by indices (*n*, *m*). The indices, *n* and *m*, denote the number of unit vectors along two directions in the hexagonal lattice of the graphene [25]. The approximate diameter of SWCNTs can be calculated from the *n* and *m* integers and the indices also determine the electronics characteristics of the nanotube.

To date, the SWCNTs have been much investigated compared to the MWCNTs due to their unique properties. For example, their band gap can vary from zero to approximately 2 eV depending on their structures that can also vary their electrical conductivity. Therefore, they can show unusual properties of metallic or semiconducting materials. On the other hand, MWCNTs have been shown to be metals with zero-gap [25].

### 2.3. Multi-Walled Carbon Nanotubes (MWCNTs)

There are two widely accepted models used to define the structures of multi-walled nanotubes such as the parchment and Russian doll model. However the Doll model is more common. The sheets of graphite are arranged in concentric cylinders, e.g., a (0,8) single-walled nanotube (SWCNT) within a larger (0,17) single-walled nanotube. In the parchment model, a single sheet of graphite is rolled in around itself, similar to roll of paper or parchment. The distance of interlayer in MWCNT is very similar to the distance between graphene layers was found to be 3.52 Å.

## 3. CNTs in Polymer Solar Cells

The two types of CNT’s, namely SWCNTs and MWCNTs, are small in diameter which is easily compatible with the properties of organic solar cells. Given that the active molecules in the systems are typically on the order a few nanometers across, these species (nanotubes and polymer base units) can readily interact potentially leading to ready charge transfer. In organic photovoltaic (OPV) device applications the preferred diameter is up to 20 nm, and the typical diameter of SWCNTs and MWNTs are in the range of 2–10 nm and 5–100 nm, respectively [26]. Both carbon nanotubes and conducting polymers possess conjugated π-systems and the nature of their electronic interaction is anticipated to occur via π-π stacking.

**Figure 1:**
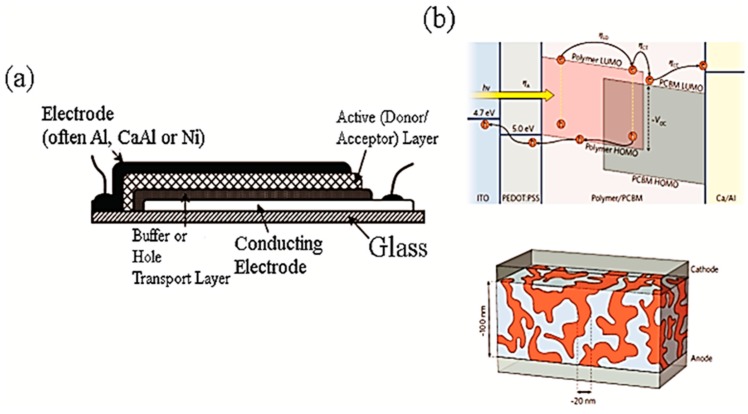
(**a**) Typical structure of a polymer based solar cell. Adapted from [34], Reproduced with permission; (**b**) The operating mechanism of an OPV with a model often presented for the network of the polymer and the acceptor. Adapted from [18], Reproduced with permission.

Choosing the corresponding material for CNT can also be very different including small molecules, oligomers, polymers, quantum dots and semiconductors (bulk and nanostructure). One of the most extensively studied structures utilized in CNT-based solar cell design are CNTs/small molecules and CNTs/polymers, where CNTs act as an electron acceptor (with some exceptions) and light is absorbed through the CNT complimentary element [27,28,29,30]. Research has been undertaken to incorporate carbon nanotubes, as a hole extraction layer in active layers [31,32] and as charge transport layer or electrode [26,33]. Here in this work the aspects of CNTs in conductive polymers are briefly discussed. A typical geometry of a polymer-based solar cell is presented in Figure 1. CNTs have been incorporated into virtually every part of the structure via a number of approaches. In Table 1 the performance of different CNT/conducting polymer-based solar cells has been summarized.

**Table 1:** Summary of Performance of CNT/conducting polymer-based solar cells.

Device Structure	J_SC_ (mA/cm^2^)	V_oc_ (V)	FF	η (%)	Spectrum (mW/cm^2^)	Ref.
Glass/SWCNT/PEDOT:PSS/P3HT:PCBM/Ga;In	6.50	0.50	0.30	0.99	-/100	[26]
Glass/ITO/P3OT/P3OT:SWCNT/Al	0.12	0.75	0.40	0.04	AM 1.5/100	[31]
Glass/ITO/PEDOT:PSS/PTEBS:MWCNT/C60/Al	1.52	0.57	0.62	0.55	AM 1.5/100	[32]
Glass/SWCNT/PEDOT:PSS/P3HT:PCBM/Ca/Al	13.78	0.57	0.53	4.13	AM 1.5/100	[33]
FTO/PBT/POT/SWCNT-TIOPH/Ca/Al	1.81			1.48	AM1.5/155	[34]
Glass/SWCNT/P3HT:PCBM/Al	4.46	0.36	0.38	0.61	AM 1.5/100	[35]
Glass/SWCNT(H2O:SDS)/PEDOT:PSS/P3HT:PCBM/LiF/Al	7.30	0.59	0.46	2.2	AM 1.5/100	[36]
PET/SWCNT/PEDOT:PSS/P3HT:PCBM/Al	7.80	0.61	0.52	2.5	AM 1.5G/100	[37]
PET/SWCNT/ZnO-nw/P3HT/Au	-	-	-	~0.60	AM 1.5G/100	[38]
Glass/ITO/MWCNT/P3HT:PCBM/LiF/Al	4.00	0.50	0.47	0.93	AM 1.5/100	[39]
Glass/ITO/PEDOT:PSS/P3HT:PCBM:MWCNT/LiF/Al	9.33	0.57	0.38	2.00	AM 1.5/100	[40]
Glass/ITO/PEDOT:PSS/P3HT:C60:SWCNT/LiF/Al	2.69	0.54	0.49	0.75	AM 1.5/95	[41]
Glass/ITO/PEDOT:PSS/P3HT:SWCNT/Al	1.93	0.58	0.42	0.52	-/70	[42]
Glass/ITO/PEDOT:PSS/P3HT:PCBM:SWCNT/Al	4.95	0.55	0.52	1.40	AM 1.5/100	[43]
Glass/ITO/PEDOT:PSS/QTF12:PCBM:DWCNT/LiF/Al	2.37	0.56	0.37	0.50	AM 1.5/100	[44]
Glass/ITO/MWCNT/P3HT:PCBM/LiF/Al	7.30	0.61	0.62	2.70	AM 1.5/100	[45]
Glass/ITO/ODA-SWCNT:P3HT:PC70BM/BCP/Al	7.66	0.52	0.44	1.76	AM 1.5/100	[46]
Glass/FTO/MWCNTs/MoO3/P3HT:PCBM/Ca/Al	8.88	0.51	0.46	2.1	AM 1.5/100	[47]
Glass/ITO P3HT:PCBM:SWCNTs/Al	11.46	0.57	0.46	3.02	AM 1.5/100	[48]
Glass/ITO/PEDOT:PSS/*f*-MWCNT/Al	11.15	0.50	0.30	1.65	AM 1.5/100	[49]
Glass/ITO/HTM/PTM10:PTM21-CNT:PCBM/LiF/Al	0.45	0.88	0.38	0.15	AM 1.5/100	[50]

### 3.1. CNTs as a Hole Extraction Layer or the Transparent Conducting Electrode

To generate solar energy, a solar cell must have an electrode that is transparent and highly conductive. Currently, the two most common materials used to meet these requirements are indium tin oxide (ITO) (preferred) and fluorine tin oxide (FTO) (less effective). A significant issue here is that indium is rare and has to be extracted from zinc and lead ores at <500 ton/year, clearly not sufficient for mass world-wide production. Additionally high quality ITO and FTO is expensive, not solution processable and rigid, all features which limit applications [37]. These are critical barriers, as any large scale production will likely require roll-to-roll type manufacturing and opportunities in flexible applications. Furthermore, as solar cell electrodes ITO and FTO are unstable in the presence of acids and bases, and it has recently been shown that their metal ions are prone to diffusing into the electrolyte thereby reducing efficiency [51].

Electrodes based on abundant elements that are easily processable and sustainable materials must be developed. CNTs are considered a promising replacement for conventional ITO because they exhibit properties such as low resistivity, a high specular transmittance in very broad spectral range from UV to MIR, superior flexibility and they can be made using simple fabrication techniques [36,52,53,54,55,56,57,58,59]. Carbon nanotubes also form sheets, often called buckypaper, with a three-dimensional topology of the mesh-like CNT network not terribly dissimilar to a bird’s nest, which allows charge collection from a large surface area, and not only from a planar interface, like from usual ITO electrodes. The high thermal conductivities which provide heat dissipation combined with stability during exposure to light make CNTs an ideal electrode material [60]. CNTs have a remarkably high level of electronic mobility and work function similar to that of the electrode which makes them an ideal candidate for the usage in the hole-extraction layer (or hole transporting layer). Due to the high work function of CNTs, they are mainly effective in hole transportation [61,62]. A great deal of research has been carried out using CNTs as a hole extraction layer in organic photovoltaics.

High transparency and low sheet resistance are two of the most important characteristics for transparent electrodes, but for CNT-based electrodes a trade-off is necessary between these two characteristics. A film of PEDOT: PSS has the potential to replace the ITO layer over large areas (cm^2^) [63]. This type of thin film is implemented in P3HT:PCBM-based bulk heterojunction solar cells and achieved efficiency of 1% [64]. Similar work done by blending SWCNTs with PEDOT: PSS showed an improved efficiency of 1.3%–1.5% [65,66]. It is difficult to assess the exact reasons for the slightly different performance as important details such as film thickness and quality of the polymer are not generally provided and will likely be different for different workers. This improvement with the presence of the nanotubes is likely due to a combined effect of an enhanced hole collection by 3-D CNT network coupled with improved transport of charge carriers into the planar ITO part of the electrode [65].

Other early work using MWCNTs with polymers used poly(*p*-phenylenevinylene) (PPV) as the conducting polymer instead of ITO but reported very poor efficiencies [67]. A simple incorporation of SWCNT at the back electrode with the PEDOT:PSS layer and P3HT:PCBM as an active layer exhibited efficiency of 0.99% on glass and 2.50% on PET substrate [37,68,69]. The effectiveness of the CNT electrode was attributed to the three-dimensional nature of the interface between the SWCNTs and the P3HT-PCBM nanocomposite. It has been reported that MWCNT on ITO yields a total power conversion efficiency of 0.93% [39] while an oxidized MWCNT can improve the conversion efficiency to 2.70% [70]. The oxidization process helped to increase the fill factor from 0.47 to 0.62 [70]. The increase in fill factor was thought to be due to an improved matching of the work function electronic states between the polymer and nanotube mixture with the introduction of the function groups on the MWCNTs [71]. This provides better overlap for improved electron and hole transportation. It is also reported that the acid oxidation treatment creates oxide layer on the CNT films and improves the conductivity as well [72,73].

It has been demonstrated that brush painting is a cost effective method of fabrication [74]. In this particular work a commercial ink is used and brushed on plasma processed PET. The plasma processing helps adhesion of the CNTs. Although the type of CNT is not specified, an efficiency of 1.63% (with *FF* 0.4049 and *J_SC_* 7.12 mA/cm^2^) is achieved. A recent report by Capasso *et al.* [47] showed that MWCNTs deposited on FTO can provide an increased efficiency of 2.1%, with improved *FF* of 0.46 and *J_SC_* 8.88 mA/cm^2^.

**Figure 2:**
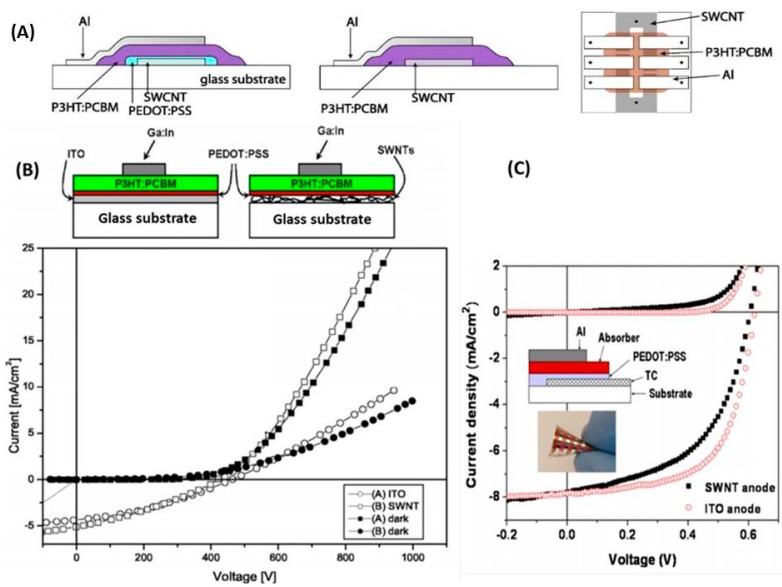
Various approaches to incorporate CNTs in the hole harvesting or conducting components of a polymer solar cell. (**A**) In first diagram, only ITO is replaced by the SWCNT layer, in second diagram both PEDOT:PSS and ITO are replaced by the SWCNT layer, while third one is sowing the top view of the device structure in which the black dots represent the points where contacts are made; (**B**) Schematic of the devices with ITO and SWNT thin film as the anodes are shown in the upper panel. The corresponding I-V curves at 100 mW/cm^2^ halogen white light and in the dark of reference solar cell on ITO-glass substrate and best solar cell using a SWNT-glass current collector are shown in the lower panel; (**C**) shows the potential flexibility using CNTs and a polymer substrate. (A) Reproduced from [66] with permission; (B) adapted from [68] and reproduced with permission; (C) Reproduced from [37] with permission from The Royal Society of Chemistry.

For bulk heterojunction devices, MWCNT-tetrasulfonate copper phthalocyanine nanocomposites have been studied since it has high work function as a hole extracting material. These devices are reported of having the efficiency of 1.25% [75]. Various approaches to incorporate CNTs in the hole harvesting or conducting components of a polymer solar cell are presented in Figure 2.

Transparent MWCNT sheets as an interlayer anode electrode for tandem OPV has been reported by Tanaka *et al.* [76]. In this work PEDOT: PSS/ P3HT: PCBM on top of an ITO coated glass substrate acts as a front cell, and the structure of the back cell is Al/Bathocuprine (BCP)/C_60_/CuPc:C_60_/CuPc/PEDOT: PSS. The MWCNT layer is sandwiched between these two front cells and back cells, thus exhibiting a device efficiency of 0.31%. However the most promising efficiency of 4.9% is obtained by incorporating a thin CNT film at the interface of P3HT: PCBM layer [62]. The report also depicts that the thin CNT film can be inserted either between the ITO and PEDOT-PSS layer or between the PEDOT-PSS layer and the active layer.

### 3.2. CNTs in the Active Layer

**Figure 3:**
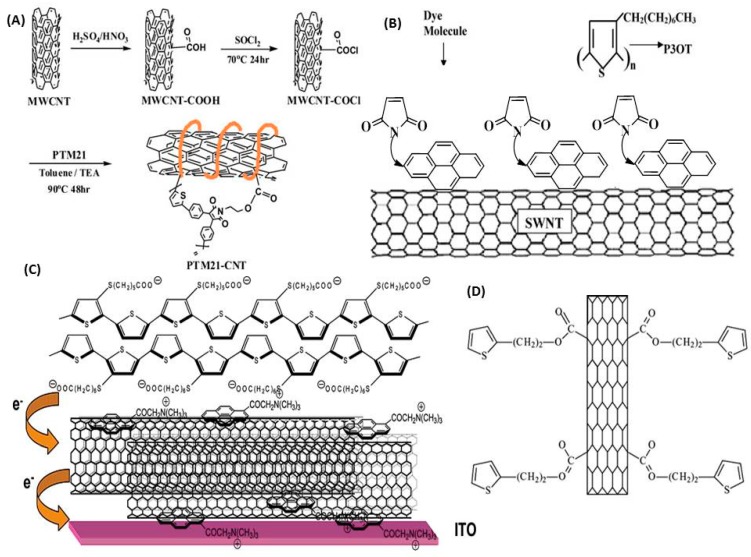
Various CNT functionalization approaches to improve incorporation of CNTs in polymer layers. Such approaches have been used both for the hole harvesting or active layers of the cells. (**A**) Synthesis scheme of PTM21-CNT, carbon nanotubes functionalized with a maleimide–thiophene copolymer; (**B**) Chemical structure of poly(3-octylythiophene) (P3OT), single walled nanotube (SWNT), *N*-(1-pyrenyl)maleimide (PM), detailed scheme of the dye molecule (PM) attachment to the SWNT surface via pi-stacking; (**C**) Schematic representation of electron transfer events in modified ITO electrodes bearing a single SWNTâpyrene+/PSCOOH sandwich layer; baselayers of PDDA and PSS are omitted from the sketch; (**D**) Schematic representation of the SWNT-TIOPH structure. (A) Reproduced from [50] with permission; (B) adapted from [78] and reproduced with permission; (C) Reproduced from [79] with permission and (D) Reproduced from [34] with permission.

Modification of the carbon nanotubes affords the opportunity to improve the dispersion of the nanotubes in the polymer matrix. There are several modifications approaches that have been used and these are summarized in Figure 3. It is important to mention that in the case of SWCNT, covalent functionalization disrupts some of the aromatic nature of the nanotubes, leaving “holes” or “defects” in the structure of the nanotube, which significantly affect both mechanical and electrical properties.

One of the earlier examples of the use of functionalization was from Pradhan *et al.* [77] who incorporated CNTs into a P3HT layer which was then used in combination with C_60_-based solar cells. The devices exhibited very low efficiencies.

Photoexcitation of a conjugated polymer, a typical light absorber in solution-processed OPV devices, generates a bound electron-hole pair called an exciton [80]. This is the key feature in OPV that requires the use of two component donor-acceptor film called the active layer. This layer performs the function of light absorption and photocurrent generation. Upon arrival of the photogenerated exciton at the interface, the heterojunction formed between polymer donor and acceptor helps to transfer the electron to the acceptor leaving behind a hole in the polymer.

Early research on CNTs used P3OT as a donor and SWCNT as an acceptor material [31,81]. This early introduction of CNTs used a simple blending approach to mix the two materials together. Although this report demonstrated a poor device efficiency of 0.02%, it introduced a new way of using CNTs in OPVs. Later, poly(phenyleneethynylene) (PPE) as a donor layer was investigated with SWCNTs as an acceptor [82]. In this case the depicted efficiency is low, however the efficiency is improved in devices yielding 0.05% efficiency [83]. poly[(2-methoxy,5-octoxy)-1,4-phenylenevinylene] (MO-PPV)/SWCNT systems are reported with nonstandard solar simulation and device efficiencies as low as 0.004% [84]. The most common used polymer with SWCNTs is the poly-hexylthiophene derivatives. These SWCNT/polyhexylthiophene-based donor-acceptor solar cells exhibited an efficiency of 0.52% [42]. Devices using modified SWCNTs have been produced through blending with poly(3-octylthiophene). An open-circuit voltage of 1.81 V is observed which is very encouraging although the PCE is still modest at 1.48% at higher than the normal light intensity of AM 1.5 [49].

SWCNTs have been used in conjunction with C_60_ as an acceptor material for P3HT bulk heterojunction devices. Recently, Schuettfort and his group reported a highly purified nanohybrid structure consisting of a SWNT coated with a monolayer of P3HT [30]. The best reported efficiency for this system is 0.57% [41]. Acid functionalized SWCNTs in bulk heterojunction P3HT:PCBM devices are also yielding efficiencies as high as 1.4%, compared to reference devices of 1% efficiency [43]. A recent study of ODA-SWCNT:P3HT:PC_70_BM as an active layer shows an efficiency as high as 1.76% [46]. More recent work has used functionalized SWCNT in photoactive layer with P3HT and [6,6]-phenyl-C61-butyric acid methyl ester (PCBM). A maximum PCE of 3.02% was obtained with an optimum 0.3 wt % SWCNTs in a P3HT:PCBM:SWCNTs device. The performance improvement was mainly attributed to the extended exciton lifetime and the rapid charge transport afforded by the nanotubes [11].

Doubled-walled carbon nanotubes (DWCNT) as an accepter material in a bulk heterojunction configuration have also been investigated. Incorporation of DWCNT with P3OT as an acceptor gives a very low efficiency of 0.001% [85]. However, when incorporated with bis-quaterthiophene DWCNT/bis-quaterthiophene donor-acceptor exhibited an improved efficiency of 0.50% [44]. Electrostatic attachment of a polyelectrolyte has facilitated the incorporation of nitrogen doped functionalized nanotubes into a P3OT layer. Nitrogen doping gives the nanotubes n-type character as opposed to the normal p-type character. The nitrogen doping did seem to enhance the performance of the devices although the performance of all the devices was poor [86]. Devices have been reported with an efficiency of 2.0% with 0.1% by weight MWCNTs in the active layer [40].

Some workers have incorporated dyes into the CNT containing layers [87]. Work with SWCNT has probed the photovoltaic properties of dye, *N*-(1-pyrenyl)maleimide (PM), functionalized single-walled carbon nanotubes (SWCNT)-conjugated polymer, poly(3-octylthiophene) (P3OT), blend composites. Addition of dye with SWCNT in the active layer has been found to improve performance. An open circuit voltage of 0.6–0.7 V was measured and thought to be due to efficient transfer of holes by dye molecules to P3OT at the dye/polymer interface and the rapid transfer of the generated electrons to the SWNTs at the dye/nanotube interface. Despite the improved short circuit current also observed, these early cells showed very poor performance [78]. More sophisticated blends of polymers have also been used for example incorporating blends of the maleimide–thiophene copolymer PTM10, multi-walled carbon nanotubes (MWCNTs) functionalized with the 2-hydroxyethyl–presenting maleimide–thiophene copolymer PTM21-OH (PTM21-CNT), and the fullerene derivative [6,6]-phenyl-C61-butyric acid methyl ester (PCBM) in various weight ratios. The PTM21-CNT behaved as an efficient compatibilizer for PTM10 and PCBM and as a charge transport assister when incorporated in the photoactive layers of the PSCs although the efficiencies of 0.15% observed for a CNT content of 0.3% are still quite low [50].

The structure shown in Figure 3C produced a donor/acceptor nanocomposite capable of 1.2% efficiencies. More promising perhaps was the fact that multiple layers of the structure provided efficiencies of over 9% which starts to move towards a regime where these cells could be commercially competitive [79].

### 3.3. General CNT Considerations

In general terms, it has been shown that beyond the electronic properties of the donor/acceptor system where functionalized CNTs are involved, the morphology also plays a key role in photovoltaic applications [88]. For example addition of functionalized CNTs to a PEDOT:PSS lowered the overall performance but did increase the current. These changes were attributed to the nanomorphology of the system [34]. Recent work has demonstrated the key importance of the nanostructure of the active layer and indeed suggests light trapping in this layer could be a powerful approach to improve performance. The best structure is difficult to predict due to the competing influences of light trapping and charge conduction [89]. Some workers have explored the effect of different types of nanotubes in polymer-based solar cell [90]. While there is a great deal of evidence proving that the introduction of CNTs improves performance, so far there is little evidence to suggest that the type of CNTs used is critical.

## 4. Conclusions

A brief review on current studies using CNTs in conducting polymer-based solar cells has been undertaken. These cells are of interest to develop future generation solar cells. CNTs are excellent materials for sunlight absorption and to generate photocarriers. CNTs have the ability to separate carriers as they can form heterojunctions with conducting polymers. In addition, CNTs can transfer electrons or holes efficiently and exhibit good PV properties. Though the efficiency of polymer solar cells is limited to a few percent, much lower than the Si-based solar cells, the commercial value of the polymer cells will be driven due to their low-cost fabrication and diverse deployment opportunities. The CNT/conducting polymer-based hybrid solar cells are still far behind other solar cell technologies. In terms of cost the CNT/conducting polymer-based hybrid solar cells would be much cheaper. However, the problem lies in the donor-acceptor material of polymer. The inherent problem of the conducting polymer seems to be the main constraint for the development of these solar cells. As a transparent electrode CNTs are very promising due to their good conducting property, low resistivity and flexibility.
